# Evidence-based communication on climate change and health: Testing videos, text, and maps on climate change and Lyme disease in Manitoba, Canada

**DOI:** 10.1371/journal.pone.0252952

**Published:** 2021-06-10

**Authors:** Laura Cameron, Rhéa Rocque, Kailey Penner, Ian Mauro

**Affiliations:** Prairie Climate Centre, University of Winnipeg, Winnipeg, Manitoba, Canada; University of Kentucky College of Medicine, UNITED STATES

## Abstract

Given the climate crisis and its cumulative impacts on public health, effective communication strategies that engage the public in adaptation and mitigation are critical. Many have argued that a health frame increases engagement, as do visual methodologies including online and interactive platforms, yet to date there has been limited research on audience responses to health messaging using visual interventions. This study explores public attitudes regarding communication tools focused on climate change and climate-affected Lyme disease through six focus groups (n = 61) in rural and urban southern Manitoba, Canada. The results add to the growing evidence of the efficacy of visual and storytelling methods in climate communications and argues for a continuum of mediums: moving from video, text, to maps. Findings underscore the importance of tailoring both communication messages and mediums to increase uptake of adaptive health and environmental behaviours, for some audiences bridging health and climate change while for others strategically decoupling them.

## Introduction

The study of climate communications has become increasingly active over the past two decades [1], which is important given that research on mitigation and adaptation are more robust than strategies for how best to engage people with this information [2]. Climate communications research unpacks the challenges for reaching audiences, including: the complexity of climate messages and lack of public scientific literacy (e.g. [3]); the public perception that climate impacts are felt elsewhere, known as psychological distancing (e.g. [4–6]); and the importance of uncertainty in climate science versus how it is perceived by the public (e.g. [2,3]). There is a growing need to emotionally connect climate change with the values of specific audiences.

One climate communications approach is to frame the issue in relation to other concerns of the audience, such as health. A public health framing of climate change has been found to shift the issue from being overly complex and distant to becoming more personal and relatable (e.g. [7,8]). For instance, Myers et al. [9] found a health frame to be more likely to generate feelings of hope and support for action on climate change than frames focused on environmental degradation or national security. By focusing on the health benefits of climate action, the need for greenhouse gas mitigation can become a positive and motivating, rather than threatening, message [9–11].

The mediums of communicating climate change is another area of active study, ranging from video games [12] and virtual reality [13] to interactive online platforms [14]. Online climate communication platforms including Climate Information Websites (CIWs) have rapidly grown as popular tools for distributing climate data to a range of actors and audiences to inform adaptation planning and decision making [14,15]. Many of these online platforms and communications are increasingly employing visualizations and storytelling, which are strategies known to evoke emotional engagement, enhance policy dialogue, and support envisioning potential climate futures [16–18]. Visualizations and storytelling–including maps and videos–can localize abstract and distant dimensions of climate change, encouraging audience engagement and dialogue [19], and help contextualize data, facts, and information within a larger narrative framework that is interesting and accessible [20].

While there has been an increase in development of online climate communication tools, less work has been done to test the user experience and response to CIWs [14,15] and climate visuals [21,22]. Understanding audience and user perceptions of climate communication approaches and platforms is critical for improving their efficacy, especially in cases where material creators and users may unknowingly have differing perceptions of how information is being interpreted, which can lead to misunderstanding, or worse, actual maladaptation [15]. There have even been calls for climate services to be “co-produced” by providers and users to ensure information needs are met and potential errors are avoided [23], while recognizing there is no agreed to methodology on how to do this [24].

The present study explores the use of visuals and health framing in climate change communication, by testing audience responses to various communication materials on climate change and the climate-affected infectious disease Lyme disease. Communication materials were created by a university-based research team responsible for a Canadian CIW–the *Climate Atlas of Canada* (www.climateatlas.ca)–to enable testing of various frames and mediums employed in climate communications. Focus groups were conducted in southern Manitoba, an area of recent establishment of Lyme disease, to test public understanding and perception of communication materials. This research offers insights for improving evidenced-based approaches to developing materials on CIWs as well as communications at the nexus of climate and health impacts more broadly.

## Methods

### Study area and context

The study was conducted in southern Manitoba, a Prairie province in central Canada, across three communities, ranging from urban to rural environments: Winnipeg (urban), Brandon (urban-rural), and Morden-Winkler and surrounding area (rural). Within Canada, the Prairie provinces including Manitoba have higher levels of climate skepticism than the national average, particularly in rural areas. For instance, in the federal riding of Portage-Lisgar (which encompasses Morden-Winkler) research has found that 45 percent of people believe that the earth is getting warmer partly or mostly because of human activity, compared to 60 percent nationally [25].

Over the past two decades, Lyme disease has emerged in Manitoba as the blacklegged tick (*Ixodes scapularis*) disease vector has become established in the province. The spread of blacklegged ticks has been attributed in part to climate change and land use changes [26,27], and future warming is expected to increase growth and reproductive rates of blacklegged ticks, as well as facilitate further range expansion [28,29]. In this way climate change is expected to increase the risk of Lyme disease in endemic areas, as well as well as bring the risk to new areas. Illness representation of Lyme disease can vary widely between individuals; common symptoms may include a circular “bulls-eye” rash around the bite area and flu-like symptoms, such as fatigue, fever, headache, and hot and cold sweats, while in the minority of cases the disease can cause serious neurological effects. In addition to the provincial and federal Lyme disease surveillance efforts to track the disease spread [30,31], there is a need to understand how best to communicate with the public about the evolving risk.

### Research design

A qualitative design based on the focus group method [32] was used to explore responses to visual and written communication materials about Lyme disease and climate change. These focus groups were divided in two parts, with the first exploring baseline public perceptions of climate change and Lyme disease, and the second exploring responses to communication materials. The present article focuses on findings from the second part of the focus groups (i.e. materials testing); results from the first part as well as a detailed description of the method are shared elsewhere [33].

**Fig 1:**
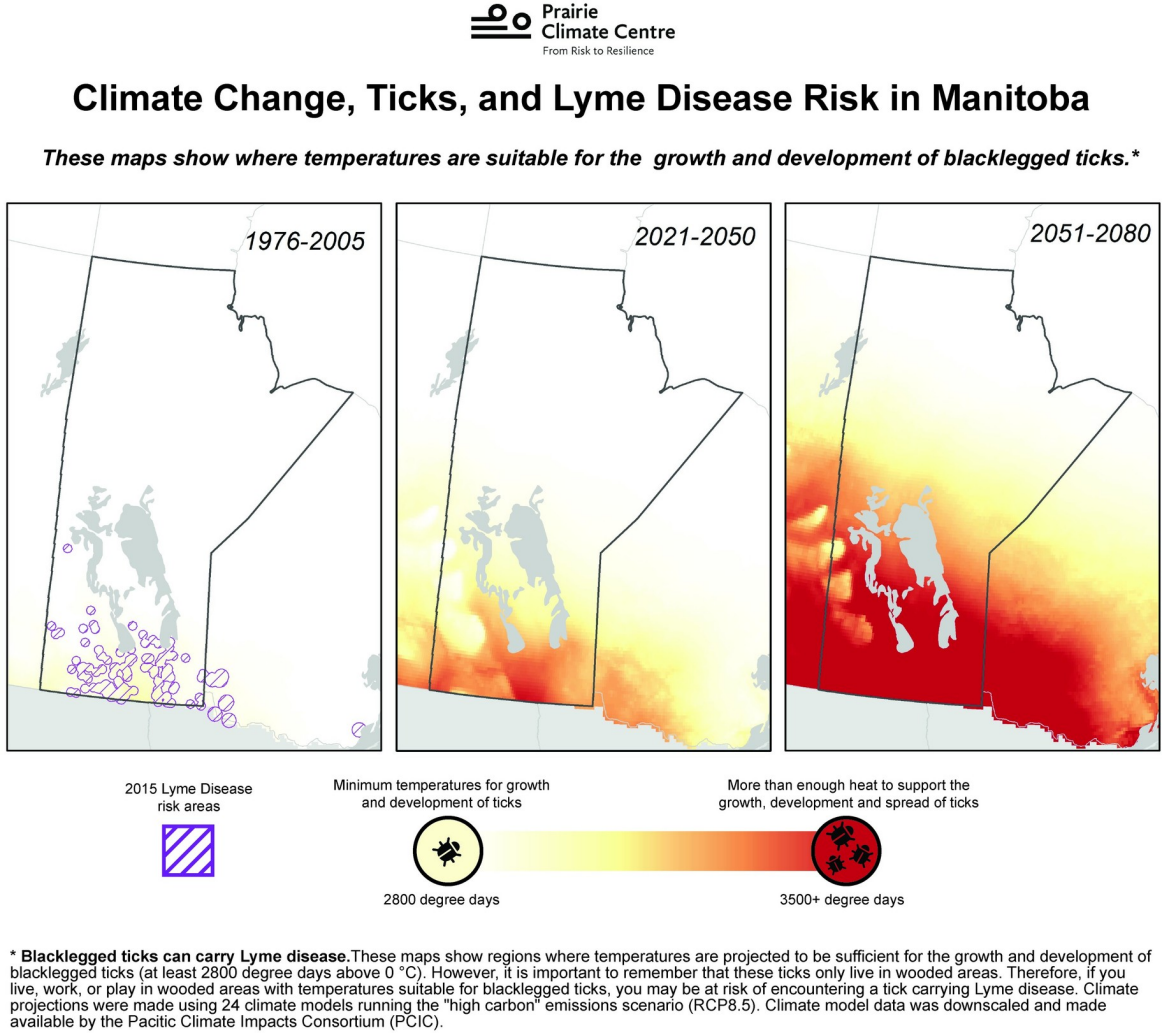
A map showing the projected spread of Lyme disease, one of the communications materials on Lyme disease and climate change that was tested in the focus groups.

To inform the creation of communication materials related to Lyme disease and climate change, interviews with six experts from the areas of Lyme disease, public health, and climate change research were conducted alongside a review of academic and grey literature. Three communication materials were developed: a short video (five and a half minutes); a plain language article (approximately 1200 words); and two series of maps illustrating the temperature suitable for the spread of the blacklegged tick (*Ixodes scapularis*) under future climate change in Manitoba and Canada (Fig 1). Materials were developed by a Canadian CIW research team in a participatory and iterative way, ensuring that experts were able to verify the accuracy of the data and information, and that they were properly represented in the video.

### Participants and procedure

This research received ethics approval from the University of Winnipeg Human Research Ethics Board. Two focus groups were held in each of the three communities over November and December 2019, for a total of six focus groups with 61 participants. The research team collaborated with Probe Research, an independent third-party research firm who assisted with recruitment and discussion moderation. Because the focus groups were specifically testing materials developed by the research team, it was preferable to have an independent moderator to avoid any potential bias. Participants of a range of demographics and concern about climate change were recruited at random from Probe Research’s panel of over 6,000 people in Manitoba. During recruitment, a short screening questionnaire was conducted by phone which asked: age, climate concern, gender, city or town of residence, ethnicity, education, occupation, and time spent working outdoors. People who had participated in a focus group at any point in the past year, or who worked for media, advertising, in the field of climate science, or at the University of the researchers, were excluded.

Participants were divided into focus groups according to level of concern about climate change as indicated in the pre-screening questionnaire, with one “high” climate concern and one “low” climate concern group per community (Table 1). The focus groups discussions were guided by a moderator, following a moderator’s guide developed by the researchers (the full guide can be found in the Supporting Information (S1 File)). Discussions were structured in two parts: (1) to explore participants’ perceptions of each of the three communications materials (video, map, and article); and (2) to allow for comparison of the materials. In the first part, the facilitator presented one material at a time and allowed participants to become familiar with it. Afterwards, the facilitator prompted participants to discuss their impressions of this first material (e.g. *“What’s the key message of this material*?*”*, *“What did you like or dislike about it*?*”*). They proceeded in the same way for the two other materials. The materials were presented in randomized order across focus groups. For the second part of the discussion, the facilitator prompted participants to discuss the comparison of all three materials (e.g. *“Which material was the most and least effective*?*”*, *“How does the focus of all materials compare*?*”*).

**Table 1:** Overview of focus group composition (n = 61). Full sociodemographic information of focus groups can be found in [33].

Group		Level of climate concern	Number of Participants	Education (attended college/uni)	Age (range, avg)
**Winnipeg**	W1	High concern	11	8	24–68, 49.3
	W2	Low concern	10	9	33–80, 56.3
**Brandon**	B1	High concern	10	7	21–65, 48.2
	B2	Low concern	8	6	42–71, 59.6
**Morden- Winkler**	M1	High concern	11	9	33–70, 51.8
	M2	Low concern	11	5	25–68, 43.5

Each focus group discussion lasted approximately 90 minutes. Written individual informed consent was obtained by participants before starting the focus group discussions, and participants were each compensated one hundred dollars for their time. All discussions were audio recorded, transcribed verbatim and checked for accuracy.

### Analysis

We conducted qualitative thematic analyses on the discussion transcripts in a qualitative analysis software (NVivo 11.4). The coding procedure was developed jointly by the four authors and two researchers independently coded all the data, with a third researcher being consulted in case of disagreements. First, we organised the segments into categories according to if they discussed a) the article, b) video, c) map, or d) the comparison of materials. Second, we identified themes describing participants’ responses to each material separately. We coded themes describing participants’ initial reactions to materials (e.g. confusion, skepticism, interest), attitude and behaviour change, and their perceptions of the key message of the material. These themes were compared across all three materials to identify similarities and differences in responses across the materials. Third, we coded the segments in which participants were explicitly asked to compare the materials, by identifying key themes emerging from the discussions. To guide our identification and interpretation of key themes, we calculated frequency counts of each theme and compared them across materials and across levels of concern for climate change (high vs. low concern, as indicated on the pre-screening questionnaire).

## Results

Overall, participants critically evaluated the materials and they expressed both positive and negative responses, and generally participants in high climate concern groups discussed more positive responses while those in low climate concern groups were more negative and critical. Themes that emerged to describe participants’ responses are presented first according to each material separately, followed by themes emerging in the discussion of comparison of materials. Differences and similarities between high and low climate concern groups are highlighted throughout.

### Material perceptions

#### Article

When reading the article, many people across all six groups expressed that the specific statistics and facts about Lyme disease were something that stood out to them, either as new information or something they thought was interesting and compelling, including: the rate of spread of blacklegged ticks per year; the increase in cases of Lyme disease; the fact that blacklegged ticks were not previously in the Prairies; and the definition of Lyme disease. When asked what the key message of the article was, the majority of people (in 4 out of 6 groups) said it was the connection between Lyme disease and climate change, while a minority (2 out of 6 groups) said it was primarily that Lyme disease and ticks are increasing, and there is a need to adapt.

Across all three low climate change concern groups, there were many people in each group who remained skeptical of the link between climate change and Lyme disease after reading the article. Many said that the article did not show proof that the rise in Lyme disease is related to climate change, while others said they simply “don’t buy it”. In one low climate concern group, someone declared that the article was “propaganda,” and in another someone noticed that amongst the peer-reviewed literature there were also news articles and grey literature cited, and said that the article was not credible because of its sources. Among those in the high climate concern groups, some people wanted more information on the implications of the connection between action on climate change and Lyme disease. As one participant in Winnipeg stated: “Do you think reducing emissions–which I agree is needed and a must–do you think it’s going to change anything about Lyme disease? Even if we stop producing any emissions tomorrow, [the ticks] are here.”

#### Video

Many participants across groups reported enjoying the video and finding it engaging. A key message identified was that blacklegged ticks and Lyme disease are not abating and thus people must learn to adapt and take preventative measures. Other key information reported was how Lyme disease is spreading, how ticks survive in warmer weather, and the increasing risk in Manitoba. As one Winnipeg participant described their key takeaway: “Lyme disease is growing a lot faster than I thought. It’s going to become more and more of a threat.” People found the visuals particularly helpful, such as the images of the bullseye rash common after a tick bite, the blacklegged ticks themselves, and researchers sampling for ticks in suitable habitat. People in several groups also commented that they liked the story element; as one Brandon participant described, “It was good how they went from… a personal story and then kind of backing it up with some of the research.” The preferred messengers from the video were the community member who had contracted Lyme disease, the medical doctor who actively sees Lyme patients, and the field researcher who sampled ticks, as opposed to the scientists in labs and offices.

People in four groups reported having more of an emotional reaction to the video, while one participant said the video had a more positive valence and “wasn’t as much doom and gloom.” When asked whether the video was about Lyme disease, climate change or both, generally the consensus was that it was more about Lyme disease. Only one group had several people comment on the link between the spreading of Lyme disease and climate change.

Many of the same skeptical critiques were brought up for the video as with the article amongst the three low climate concern groups. Many people across all three groups mentioned that there was not sufficient proof that the tick spread was related to climate change, though people were not able to articulate the additional information they would require. Many people restated their original views, saying things such as “animals are just moving,” “the weather hasn’t changed that much” or “it is a cycle.” A few people admitted that they would not watch the video if they came across it because they did not find it interesting or informative.

#### Maps

Participants had a more negative reaction to the maps overall. Across all six groups, people identified the key message of the maps was that temperatures are getting warmer and things are going to get worse. Some people pointed out that this will lead to an increase in the spread of ticks and Lyme disease, but for many that was not immediately clear. Generally, people agreed that the main message of the map was that climate change is getting worse, while several said it was the link between climate change and Lyme disease. People in several groups commented on the intensity of the red colouring, with one participant commenting, “When you make the province of Manitoba look like it’s wearing a Calgary Flames [hockey] jersey, it looks scary.”

There were many criticisms of the maps, with people in every group saying that the language used in the map caption was too complex and technical for an average person. Participants expressed not understanding climate models and projections, degree days, high carbon emissions scenarios (RCP 8.5), and down-scaled climate data. Another point of confusion was the timeframes of the past, near future, and far future used on the maps; many people in five of the groups wanted to see the present represented (i.e. 2019). People in two of the groups mentioned that the map of the far future (2051–2080) had no effect on them because it was not relatable. One person mentioned, “…by 2080, I’m sorry, I’m not going to be around. How can I relate to that last picture?” Additionally, some people in three of the groups took issue with the use of climate projections, suggesting that they are not accurate or reliable. One person described scientific projections as “guessing”. As another person mentioned, “Well…it’s such a huge timeframe and as I said, you look at the history of forecasting weather… they’re accurate to a degree, but never exact. And so, I look at this and okay, well, yes, they are true to a degree, but how accurate?” A few people across two of the groups simply did not understand the map at all, with one stating “It’s a pretty picture. It looks like it’s getting worse, but we don’t understand why.”

There was also mention across three groups (of both high and low climate concern) that the map was “fear mongering” or trying to scare people by making the changes look extreme. One person in Winnipeg even suggested that Lyme disease was being used to incite fear about climate change: “It makes me think more that these [researchers] are just using Lyme disease to scare you about climate change.”

A summary of common strengths and criticisms for the three materials are found in Table 2. Criticisms and recommendations were more commonly discussed because of the design of the focus group discussion and moderator’s probes for feedback.

**Table 2:** Summary of common strengths (+) and criticisms and recommendations (-) of each material and comparison across materials for low concern (W2, B2, M2), high concern (W1, B1, M1), and all groups.

	Low climate concern	High climate concern	All (Low and High concern)
**Article**	− Credibility of some sources not trusted − Not enough proof of climate change impacting Lyme − Want more “balance” and more information on other factors influencing Lyme spread	− Too simplistic − Too much focus on individual action, not enough on systemic solutions − Want more information on the implications of acting on climate for the spread of Lyme − Want more visuals	+ Lyme disease statistics (e.g. rate of movement of ticks, reported cases) + Information about tick species and hosts − Unclear language − Want more stories, lived experiences − Want more info on Lyme symptoms
**Video**	− Not enough evidence of climate change − Want more info on other contributors of Lyme disease spread	+ Positive message + Personal story − Lacking key visuals (e.g. pets) − Too long − Lab-based scientists are less interesting − Not enough call to action	+ Practical visuals (e.g. bullseye rash, tick species) + Animated map (showing spread) + Doctor, field researcher, layperson with lived experience were best messengers − Want more information on layperson’s story and long term effects of Lyme Disease − Want more info on different stages and species of ticks
**Map**	− Projections not credible − Would be better with the US included to previous disease range	+ Interesting info on climate + Good at communicating alarm and urgency on climate	− Technical language (e.g. “degree days”, “high carbon scenario”) − Confusing gaps in time frame; far future is not relatable − Concept of climate models is unclear − Colours are too alarming − Risk areas overlaid on projections are confusing
**Across all three materials**	− Want more information on Lyme disease and less on climate change, separating the issues	− Want more information on solutions for climate and Lyme	+ Most people found video most engaging of the three materials, it created emotional engagement, and connected with the audience − Materials are too long, should be shortened and simplified

### Materials comparisons and recommendations

#### Materials elicited a range of responses

When asked which of the three materials had the most impact or was the most effective, the majority of people in five of the six groups clearly felt that it was the video. Across four groups, people mentioned that they liked the video better because they were audio-visual learners, while others in two groups said it had more of an impact because there were “real people” in the video. Interestingly, even the more climate skeptical participants preferred the video, as one Brandon participant explained: “I’m not necessarily a hundred percent onboard with the scientific data that you’re spelling out there, but the anecdotal situations on how somebody actually contracts the disease, and to look for that, hits me a lot better.” Several people suggested the video would be the best medium to reach younger audiences who are online frequently, though it was suggested that the tool needed to be shorter in length.

A few people in two groups said that the article was the most effective material, explaining that they generally enjoy reading so they were most drawn to this format. The map was most commonly identified as the least effective material. This was brought up across all three of the low climate concern groups, with many claiming that it was missing data, was “fear-mongering,” “alarmist,” or looked like “propaganda,” with one participant even saying it should be “thrown in the garbage.” Compared to the video, the map was seen as less credible and believable.

#### Limited attitude and behavioural change

When asked if any of the materials changed their attitudes, across all four groups outside of Winnipeg, many people mentioned that none of the materials changed their attitudes towards Lyme disease, climate change, or the link between them. In some cases, this was because they expressed already having this knowledge, while for others the information was not sufficient to convince them of the link between Lyme disease and climate change. One person in a low climate concern group mentioned that they became less worried about ticks after watching the video.

Others reported that the materials did change their attitude towards the issues, by making them more aware of the growing risks. For these participants, there was a range of responses to whether they would change their behaviours as a result of their shift in attitude. Some said they still did not perceive a big enough risk from Lyme disease to change their behaviour, while others said they would take more precautions against tick bites, particularly with children and pets. Similar to attitudes, many people, particularly in rural areas, expressed already having preventative habits, such as checking for ticks, dressing accordingly, and using bug spray.

#### Confusion regarding the purpose of materials

The materials were commonly criticized for having an unclear purpose or “muddled message.” In four groups, participants were confused about the purpose of the materials, whether it was to motivate people to act on climate change or to adopt preventative behaviours in response to the increasing health risks from blacklegged ticks. As one participant articulated: “What’s the purpose?… Are you trying to activate people on a climate change question, or are you trying to activate them to take better care of themselves health-wise?”

People from high and low climate concern groups suggested either separating the Lyme disease and climate change messages, or prioritizing one so that there is a clear focus. Multiple people said that if Lyme disease is the focus, then climate change should be excluded or minimized in the materials so as not to lose interest from climate skeptical audiences. A participant from one of the high climate concern groups explained: “I think most of us here agree with climate change, [but] a lot of people out there don’t, so as soon as you try to frame this as ‘you better be scared of climate change because of ticks’ you’re going to have people ignore your information on ticks.” Others noted that if climate change is the main focus, then the materials should focus on other impacts because “there’s bigger issues than…ticks with climate change”. A few people argued that it is important to discuss both Lyme disease and climate change together to give context to why the ticks are spreading and increase awareness of climate impacts. As one person expressed, “I think it’s helpful that the climate change part of it is in there…it helps us understand why it’s increasing or where it’s coming from.”

#### General recommendations for improving and sharing the materials

While offering critical feedback on the materials, participants were also generally supportive of the study and gave recommendations on these communication tools to improve their efficacy and uptake. In general, many suggested clarifying the purpose and making the materials shorter and simpler, while several people suggested having more personal stories that make the content relatable. Some people wanted to see more data and “proof” while others wanted less scientific information. Other recommendations were to include: more information on solutions (e.g. how the general public can do their part to take action against climate change and slow the spread of blacklegged ticks); more comparative visuals of different types of ticks; more details from the story of the Lyme-affected community member; more information on the long-term effects of Lyme disease; and more “relatable” characters in the video.

Suggestions for where to share the materials included: in outdoor magazines, hunting and fishing guides, hardware stores, a provincial park office or campground, schools, doctor’s offices and health centres, nature TV shows, weather websites, or online advertisements. Other suggestions included creating an app or online game for kids, adding tick information to google maps, or sharing it on social media. There was a suggestion to have the materials somewhere people are “forced” to watch or read. Despite the constructive criticism regarding the materials, many focus group participants had thoughtful ideas on how the tools could be maximally shared.

## Discussion

In this study, we explored responses to three types of communication materials–video, maps, and an article–about the connection between climate change and Lyme disease. We identified three key findings, as discussed below.

### Mediums of communication: Emotional engagement through video and story

Most participants expressed a preference for the video, as it related visuals and stories. This finding aligns with previous research which suggests that visuals can make climate change impacts more concrete, relatable, and engaging (e.g. [21,34,35]) and further suggests that video in particular can change public attitudes on climate change (e.g. [36]). Video allows for dynamic storytelling in a way that static visuals [37] and text alone [34] do not. While the literature is divided on whether climate visuals of impacts or solutions are more effective [38,39], the medium of video can allow both impact and action-oriented visuals to be woven together to produce a message that provides a balance of hope and urgency to motivate viewers. Similarly, some have argued that effective climate imagery should capture the depth and complexity of both the problem and its solutions to engage a wide audience [22,39]. The results here suggest that both visuals of the problem (e.g. animated map of disease spread) and the solution (e.g. scientists conducting field surveillance) resonated with participants.

The video was found to be a more effective medium for engagement because of its use of narrative and focus on characters, such as the community member who shared his story of contracting Lyme disease. While some studies have emphasized the importance of featuring “normal” people in climate communications (e.g. [40]), in practice there is often a lack of human stories [22]. Story narratives are found to be better than fact-based narratives at facilitating experiential processing and motivating action-taking on climate change through heightened affect and emotion [18,41]. These results align with this previous research which argues for emotional anchoring of climate information (e.g. [42]), while also raising interesting questions around perceived credibility in storytelling on climate change. The fact that a non-expert messenger sharing their experience was more engaging for many–and more believable for some–than experts describing information fits with previous research on the importance of a new relationship between lay and expert-based knowledges on climate change, especially concerning a doubtful public [43].

At the same time, the other climate visuals tested–the maps–were found to be the least favoured and effective of the three materials, with the combination of technical language, climate model projections, carbon scenarios, and red colouring leaving participants confused or skeptical. Maps have become popular in communicating scientific information on climate change over the past decades, as they can be particularly effective when hazards have a spatio-temporal component [44]. However, the use of scientific images in climate communications (e.g. maps, graphics) often depict climate change as a natural process and may not compel individual action, thus leaving viewers feeling powerless [45]. In our study, people found the maps alarming or impactful, few understood them fully, and many expressed negative feelings towards them as a result. Research on climate information websites designed to share downscaled climate data have found similar results, as these sites often assume a higher level of understanding of scientific concepts and associated jargon among their users than there actually is [15]. Another common critique among participants related to the “colour ramp” of maps, which people felt was scary, confusing, or deceiving. Colour ramps portray not only the data but also evoke different emotional reactions, carrying cultural associations such as red for danger [37,46], and these various design choices can trigger differentiated responses that affect the perceived credibility of climate maps and visualizations [47]. Compared to the video, the more complex mapped climate projections were not as comprehensible for the vast majority of participants. While the medium of video, and to a lesser degree the article, translates climate science through narrative and emotion, climate projection maps did less to translate the knowledge. Arguably, a more sequenced approach for climate communication when engaging audiences of low knowledge or concern on climate change might be: (1) video that incorporates storytelling as a tool for initial engagement; (2) articles with narrative and visual components as a second step; and (3) more complex climate maps once the audience has been engaged. Maps are a critical tool to communicate scientific information, but may require more audience knowledge and participation in decoding the information, and are perhaps most effective in combination with other climate visuals and contextual information.

### The need to clarify the message: Health or climate?

A key finding relates to the confusion about the main message and purpose of all communication materials. Many participants were unsure if the purpose of the materials was to promote action to mitigate climate change or to encourage adaptive behaviours in response to the increasing risk of Lyme disease, and most suggested choosing one or the other would make the materials more effective. This seems to challenge the emerging climate communications literature that suggests that health is an important frame for effective dialogue with an increasingly polarized public [9,48,49]. Indeed, public health communicators are also developing best practices for communication around the health effects of climate change [50].

**Fig 2:**
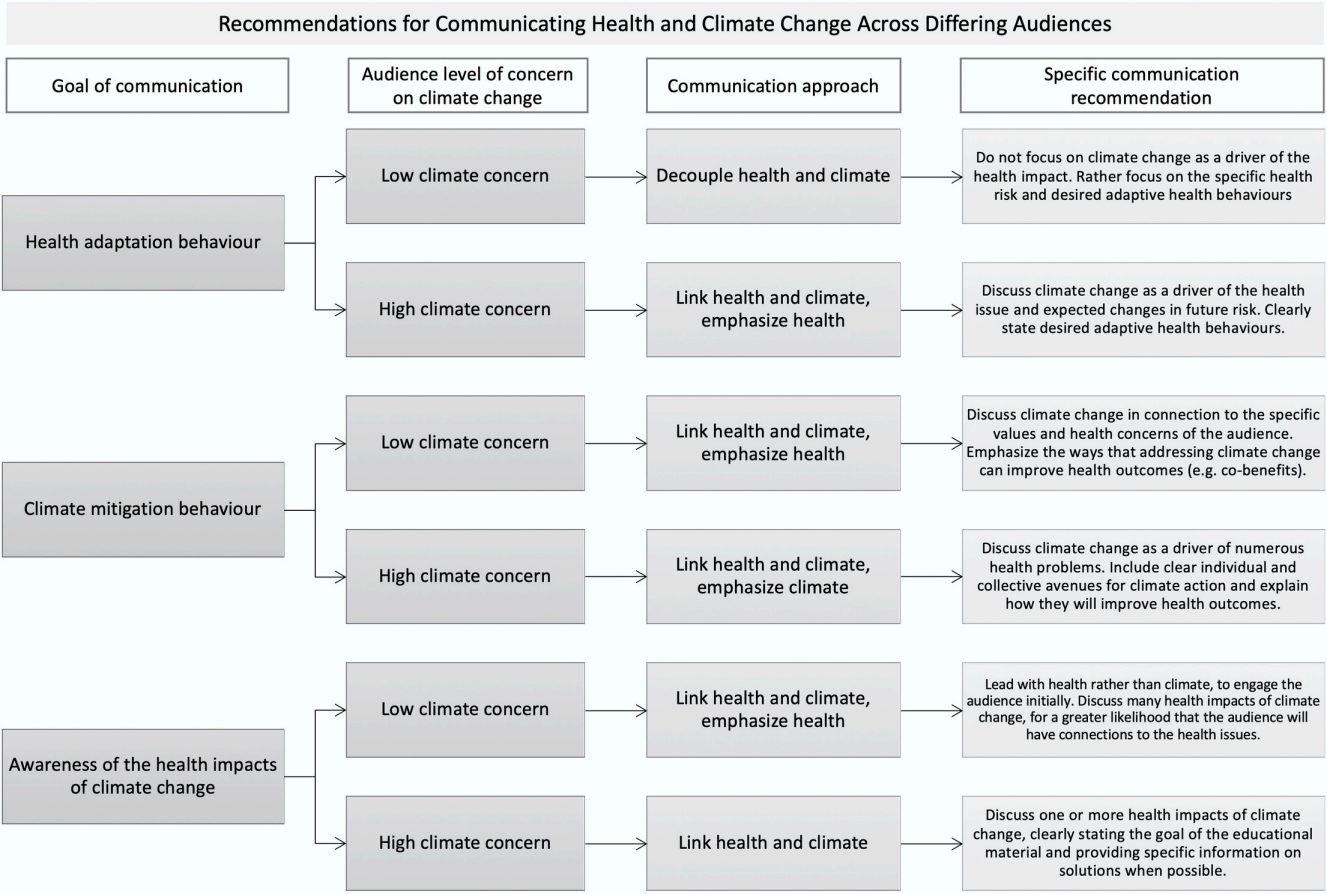
Recommendations for communicating health and climate change across differing audiences.

The results here suggest that marrying public health and climate change information is not as straightforward as it may seem, as the association of the two topics may draw skepticism from those already unconvinced or skeptical of climate change. In this context, it seems that “strategically decoupling” the two issues in communications might be more effective in some cases, especially if the primary goal is to motivate adaptive health behaviours among skeptical or mixed opinion audiences [51]. However, if the goal is to increase awareness regarding the human dimensions of climate change, reframing climate change from an environmental to a human health issue is essential. At present, there is a need to critically evaluate what a health frame in climate communications looks like, and these results suggest a broader approach that has the widest connection to the health of the target audience is likely most effective (i.e. a focus on Lyme disease alone was not convincing for many). In this regard, health officials and climate communicators may approach and present the content differently–by separating or connecting climate and health risks–according to their respective goals and audiences (Fig 2).

### The challenge of moving from attitudinal to behavioural change

The final key finding shows that materials were interpreted differently by participants based on their previously-held beliefs and cultural values, and that self-reported attitude and behaviour change was limited. These results are consistent with previous research [52–54] and may be explained by the high social costs and barriers of updating and changing one’s beliefs [54]. Specifically, those already skeptical of climate change often expressed skepticism about the communication materials and brought up other factors that are affecting the spread of ticks, or shifted the conversation to an unrelated topic. Yet, many of the participants who believed in the risks were not willing to change their behaviour. Most participants said they would not take extra precautions to prevent tick bites after seeing the materials, either because they do not perceive the risk as great enough or because they already enact these behaviours. Aenishaenslin et al. [55] found similar results in a national survey following a Lyme disease communication campaign by the Government of Canada, showing high awareness of Lyme disease but low rates of adopting preventative behaviours. Indication of participants adopting behaviours to mitigate climate change after seeing the materials was even lower, indeed, no participants said they would change their behaviours to act on climate change. The shift from attitudinal change to behavioural change is a well-known challenge in psychological research, and communication materials must stimulate deeper engagement with the issue, particularly through an emotional connection, which seemed to best demonstrated through the use of video in our study.

### Adapting the materials and implications for other CIWs

Based on the findings of this study, the tested communications materials were revised based on participant feedback to increase their efficacy, and were ultimately published on the *Climate Atlas of Canada* to support evidence-based content development of the climate information website (CIW) [56]. While the participant responses here provide insight for CIW content development, the process of materials testing and adaptation also provides insight into an approach for future evidence-based materials development in climate communications. Changes made to the materials and considerations and recommendations for CIWs are summarized in Table 3.

**Table 3:** Summary of changes made to the materials and recommendations for future online climate communications based on current findings.

	Article	Video	Map
**Changes made to the materials in response to participant feedback**	✓ Clarified technical language ✓ Included additional images to accompany the text ✓ Added more information on Lyme disease symptoms and illness representation ✓ Added more information from the story of the person with lived experience of the disease	✓ Added more of the messengers that participants found most engaging (e.g. person with lived experience and medical doctor) ✓ Added specific visuals that participants requested (e.g. comparisons of different tick species, pets, and tick habitat)	✓ Changed the colour ramps to use less red and make the colours less bright ✓ Eliminated technical terminology and simplified the language in the map caption and legend ✓ Changed the language around the time periods shown and removed the 2015 Lyme disease risk areas ✓ Added a map to visualize a lower carbon pathway (RCP 4.5) to show how climate action could lessen the spread of Lyme disease
**Recommendations for CIWs and considerations for future climate communications research**	➢ Continue to refine best practices for using simple language to communicate complex climate impacts ➢ Attention to which sources (academic or non-academic) are most credible to different audiences	➢ Create different length videos to reach different audiences ➢ Include more relatable messengers, particularly those with lived experience of health impacts ➢ Explore collaborations for sharing content across platforms to reach different audiences	➢ Attention to emotions evoked by certain colour ramping on climate maps ➢ Accompany climate visualizations such as map projections with information on how to interpret ➢ Embed maps in other materials where they can be contextualized for unfamiliar audiences
	• Creative, visual mediums of communication are most effective, but diversity is important. Incorporating elements of storytelling and relatable visuals into communication products are most effective at engaging people. • Climate communicators should explore how to better integrate content into platforms and contexts that are already relevant to audiences (e.g. share information through outdoor magazines, parks, hunting and fishing guides, TV commercials, doctors’ offices, etc.). • Climate communicators should be reflective of when and how maps of climate projections are and are not useful for communicating to lay audiences versus professional users. There is a need for further user testing of maps and other climate visuals created for CIWs.		

Not all feedback was incorporated in the revised materials, in part due to limitations of climate model data and visualizations, but also because of opposing feedback from participants. In alignment with previous research, the results here show that different audiences along the climate concern spectrum require different materials and framing, and thus user-testing can help refine materials to increase their efficacy across audiences. This might also help avoid counterproductive communication frames; for example, in our study some participants had their skepticism reinforced by materials instead of creating engagement with the topic. Finally, the platform and context within which content is communicated should be taken into consideration and material should be adapted accordingly (e.g., have the information available in contexts other than a climate information website for skeptical audiences).

### Limitations and opportunities

A limitation of this study–inherent to focus group methods generally–is the potential for certain people to dominate discussion or influence others’ comments, depending on the social dynamics that emerge in the group. This was considered and steps were taken by the facilitation team to ensure that everyone participated as equally as possible. The discussion was also designed to probe for feedback on the materials, which may have led to there being more negative than positive comments. While the study was regionally-specific, perhaps limiting their applicability outside of this area, the results also clearly demonstrated the unique challenges of communicating climate change in an area known for skepticism like the Canadian Prairies [25]. Future research should explore public perceptions of communications materials in different regions in Canada and with different issue-specific or more general health framings of climate change, to further articulate and inform effective communications on the full range of health impacts of climate change.

## Conclusion

The intersecting crises of climate change and public health demand communication approaches that bolster public engagement in these issues for urgent and far-reaching action. The findings from this focus group study in central Canada shed light on benefits, challenges, and considerations in communicating about the relationship between climate change and climate-affected Lyme disease, with implications for the use of different mediums and frames within climate communications. The results illustrate the efficacy of the use of video as a climate visual, while another visual material, a series of climate maps, was less effective and less understood across audiences. It was clear that the video successfully translated scientific information through narrative elements, visual storytelling, and relatable messengers, while the climate maps required prior audience knowledge and ability to engage with more complex scientific information. Based on the results here, we suggest video, and to a lesser extent plain language articles, may be better mediums of communication for audiences with lower levels of knowledge or concern on climate change, which may prime these individuals and communities for subsequently interpreting more complex materials such as climate projection maps.

Additionally, the findings underscore the importance of tailoring the communication frame to the specific purpose and audience of the climate and health communications. For example, despite creating materials aimed at inspiring adaptive health behaviours, these interventions did not resonate with audiences of low climate concern, and thus it may be best to decouple climate and health issues in this case. In the first stage of this research, we developed an exploratory model regarding the relationship between Lyme disease and climate change risk perceptions, which may further assist those interesting in framing and messaging on these topics [32]. Whereas, if the goal is to initiate action on climate change or change public opinion on climate change as a health issue, an approach that links climate and health and is tailored to the audience levels of climate concern is key.

This research is among the first to test audience responses to climate change visualizations in Manitoba, and more research is needed to further test tools such as these from this and other CIWs. It illustrates the importance of user-driven, evidence-based approaches to climate change communication and associated development of CIWs, with a strong, continuous feedback cycle between developers/users and theory/practice. This paper demonstrates that a continuum of climate communication materials–from video, text to maps–may increase salience and emotional response with each subsequent intervention. Indeed, it is paramount that climate communicators evaluate, interrogate, and revise their approaches and tools to ensure information is appropriate for different audiences and their concomitant worldviews. By taking this type of evidence-based approach, climate communicators and CIW developers will ideally increase both climate knowledge and action across various audiences within society.

## Supporting information

S1 File Focus group moderator’s guide. (DOCX)
