# Supplementary material for: Evidence-based communication on climate change and health: Testing videos, text, and maps on climate change and Lyme disease in Manitoba, Canada
Source: PLoS One. 2021 Jun 10;16(6):e0252952. doi: 10.1371/journal.pone.0252952 (PMC8191974; doi:10.1371/journal.pone.0252952)
Supplement: S1 File — (DOCX) [file pone.0252952.s001.docx]

**S1. Focus Group Moderator’s Guide**

*Note: This Moderator’s Guide was developed for the use in six focus groups on Lyme disease and climate change in southern Manitoba. Questions were used to prompt discussion, but the discussion was also allowed to move to other themes as they arose through conversation.*

Welcome, Introductions, Project Background, Consent: 15 mins

- Moderator introduces herself and Probe Research.
- Explain how the group works – discussion should be fun and relaxed.
- Tonight, we’re going to have a chat about a few things, then I want to show you some materials - some visuals that I want your opinion on.
- But before we get into the details, I just want to tell you a bit about the project and the consent form and make sure we’re all comfortable.

Project background for participants:

- This research is being conducted by the Prairie Climate Centre at the University of Winnipeg, with the support of Probe Research.
- The objective of this project is to explore health impacts of climate change. Specifically, this session is part of a series of focus groups being conducted in southern Manitoba to explore risk perception around climate change and Lyme disease.
- Your participation in this focus group is voluntary and you will be compensated for your time, as discussed prior. You may withdraw your participation at any point in this session, should you choose. We guarantee that all participants will remain anonymous in any results shared publicly. We also ask that you keep everything you hear today confidential for the privacy of all participants.
- The session is being audio recorded and will be transcribed and analysed to identify themes. Audio recordings will be stored securely in the researchers’ lab at the University of Winnipeg, and will never be released publicly.
- Results from this discussion may be used in research publications in academic journals, at conferences, and/or on the PCC’s website.
- Colleagues from the UW are listening in.
- All of the information I just shared is written on this consent form. We ask that you review and sign this form if you consent to participating in this research.
  - Did anyone have any questions about the consent form or this research?

Housekeeping notes

Ice-breaker: Please tell me your first name only and a little about something

Discussion: Risk Perception of climate change and Lyme disease (15 mins)

To start off, I want us to brainstorm a list. When I say the phrase “climate change” - what comes to mind? It can be anything…

Follow up with...

- Do you see climate change as a risk to you personally? In what way?
- How big is this risk?
- How about to your community? In what way?
- How big is this risk to your community?

OK, how about Lyme disease - what are some of the first things that come to mind when I say Lyme disease?

Follow up with...

- When did you first hear about Lyme disease? Have you seen anything or heard anything about it lately? Where was that?
- Do you know anyone with Lyme disease?
- Do you think there is a risk of Lyme disease in Manitoba? How big is this risk? Is it getting bigger or smaller?
- How do you prevent Lyme disease? What are the things you’re supposed to do?
- Do you think Lyme disease is connected to climate change? In what way?

Communication materials presentation

I’ve got some materials here I want to show you and get your thoughts on. We’ll go one-by-one.

The first one is: *[RANDOMIZED MATERIALS]*

1. Short article to read (printed)
2. Video - 6 minute video on LD and CC
3. Static regional and/or national maps - on screen

Article - Discussion (5 mins to read, 15 mins to discuss)

I’ve got an article here for you to read on your own. While you’re reading it, I want you to do two things:

1. With the green highlighter, I want you to highlight everything that’s new to you, stuff you didn’t know - green is “news to you.”
2. You’ve each got one dot. At the end, when you’re done reading, put that dot next to the most surprising thing, the thing that had the most impact on you.

I’ll give you about five minutes with this, and then we’ll discuss….

Follow up with...

- What’s the key message of this article, if you had to put it into words?
- Did this seem trustworthy or credible? Why/why not?
- Was it clear? Or confusing? What parts?
- How did your understanding of Lyme disease change - if at all?
- Do you think you’d change your behaviour because of this article? In what way?
- Where would you expect to see this article? What’s the best way to get it in front of you? Would you actually read it? What would motivate you to read it?
- Is there anything that could be done to improve this article?

Video - Discussion (5 mins to watch, 15 mins to discuss)

Now, I’ve got a short video to show you. We’ll just watch it once and then we’ll chat about it…

- What’s the key message of this video, if you had to put it into words?
- What was one thing you learned you didn’t know?
- Did you connect with the video emotionally? If so, how?
- How did your understanding of Lyme disease change - if at all?
- Do you think you’d change your behaviour? In what way?
- Is there anything the university could do to improve this video?

Maps – Discussion (3 mins to study, 10 mins to discuss)

Now, I want to show you some map(s). You can get up and study these a little closer if you want. I’m going to give you a couple of minutes to really have a look at these…

- What’s your key takeaway from these maps?
- Did they seem credible or not? Why/why not?
- Were they clear? Or confusing? What parts?
- Did your understanding of Lyme disease change? If so, how?
- Do you think you’d change your behaviour because of these maps? In what way?
- Where would you expect to see these maps?
- Is there anything the university could do to improve these maps?

Comparison of Materials and Closing - 10 mins.

- Thinking across the three options, did the different approaches to communication impact you differently?
- Do you see value in each approach? If so/if not, why?
- Any final thoughts? If you would prefer to write any thoughts or reflections you have you can do so on the paper provided and leave them on your tables for us to collect afterwards.
